# Changes in rate and socioeconomic inequality of cervical cancer screening in northeastern China from 2013 to 2018

**DOI:** 10.3389/fmed.2022.913361

**Published:** 2022-10-05

**Authors:** Yaqian Liu, Jing Guo, Guangyu Zhu, Bo Zhang, Xing Lin Feng

**Affiliations:** ^1^Department of Health Policy and Management, School of Public Health, Peking University, Beijing, China; ^2^Department of Computer Science and Statistics, University of Rhode Island, Kingston, RI, United States; ^3^Department of Neurology and ICCTR Biostatistics and Research Design Center, Boston Children's Hospital, Harvard Medical School, Boston, MA, United States

**Keywords:** cervical cancer screening, inequalities, organized screening program, rural areas, China

## Abstract

**Objective:**

Cervical cancer, the fourth leading cancer diagnosed in women, has brought great attention to cervical cancer screening to eliminate cervical cancer. In this study, we analyzed two waves of provincially representative data from northeastern China's National Health Services Survey (NHSS) in 2013 and 2018, to investigate the temporal changes and socioeconomic inequalities in the cervical cancer screening rate in northeastern China.

**Methods:**

Data from two waves (2013 and 2018) of the NHSS deployed in Jilin Province were analyzed. We included women aged 15–64 years old and considered the occurrence of any cervical screening in the past 12 months to measure the cervical cancer screening rate in correlation with the annual per-capita household income, educational attainment, health insurance, and other socioeconomic characteristics.

**Results:**

A total of 11,616 women aged 15–64 years were eligible for inclusion. Among all participants, 7,069 participants (61.11%) were from rural areas. The rate of cervical cancer screening increased from 2013 to 2018 [odds ratio (OR): 1.06; 95% confidence interval (CI): 1.04–1.09, *p* < 0.001]. In total, the cervical cancer screening rate was higher among participants who lived in urban areas than rural areas (OR: 1.20; 95% CI: 1.03–1.39, *p* = 0.020). The rate was also higher among those with the highest household income per capita (OR: 1.30; 95% CI: 1.07–1.56, *p* = 0.007), with higher educational attainment (*p* < 0.001), and with health insurance (*p* < 0.05), respectively. The rate of cervical cancer screening was also significantly associated with parity (OR: 1.62; 95% CI: 1.23–2.41, *p* = 0.001) and marital status (OR: 1.45; 95% CI: 1.15–1.81, *p* = 0.001) but not ethnicity (OR: 1.41; 95% CI: 0.95–1.36, *p* = 0.164).

**Conclusion:**

Cervical cancer screening coverage improved from 2013 to 2018 in northeastern China but remains far below the target 70% screening rate proposed by the World Health Organization. Although rural-urban inequality disappeared over time, other socioeconomic inequalities remained.

## Introduction

Cervical cancer is the fourth leading cancer diagnosed in women globally, being responsible for ~311,000 deaths in 2018 alone worldwide (1), with >85% of the burden from cervical cancer existing in low-income and middle-income countries (2). Annually, China contributes ~18.6% of new cervical cancer cases and 19.3% of the deaths caused by cervical cancer (3). However, some cases of cervical cancer may be preventable. Recently, the World Health Organization (WHO) proposed the “90–70–90 movement” toward 2030 (4)—that is, 90% coverage of human papillomavirus (HPV) vaccination, 70% coverage of screening twice in a lifetime, and 90% access to the pre-invasive lesion and invasive cancer treatments (2).

Worldwide, two major types of national-level cervical cancer screening programs, organized programs and opportunistic programs, have been implemented by various countries to eliminate cervical cancer (5). Organized programs are supported by the government and invite all eligible women to undergo cervical cancer screening delivered by trained physicians in appointed facilities (6, 7). In an organized program, cervical cancer screening is usually paid for by the government. In contrast, an opportunistic program provides cervical cancer screening when individuals request the screening or their health care providers recommend the screening (8). Previous research has suggested that organized programs may achieve greater coverage of cervical cancer screening and may be more effective than opportunistic screening programs (7). However, there is a lack of consistent conclusions about whether organized screening can eliminate the socioeconomic inequality of cervical cancer screening. Further, no studies have investigated the impact of organized programs and opportunistic programs in low-income or middle-income countries.

To reduce the health care burden brought on by cervical cancer, especially that among residents in rural areas, in 2009, China launched an organized program called the “National Cervical Cancer Screening Program in Rural Areas” (NCCSPRA) (9, 10) to provide cervical cancer screenings to rural women aged 35–59 years. The program was subsequently expanded to cover rural women aged 35–64 years in 2012 (11). The NCCSPRA program was the very first effort made by the Chinese government to improve access to cervical cancer screening for residents in rural areas and represented a step toward the nationwide provision of cervical cancer screening (11). Staff in township health care centers provides education and mobilization on cervical cancer screening for eligible women in their jurisdiction. Women who agree to undergo screening tests are organized and transported to the appointed health care center for the examination. Yet, such a program has remained unavailable in urban areas, although residents in an urban area who are formally employed may take uniform cervical cancer screening tests provided by their employers, while other women in urban areas without this type of access may take the tests ordered by their health care providers as needed. Despite the target coverage rate of 70% for cervical cancer screening, from 2009 to 2011, only 7% of rural women aged 35–59 years were covered by organized cervical cancer screening programs in China (10). The overall screening rate among women >18 years of age was only 19.7% in 2010 in China (12, 13). No study has investigated the impact of the NCCSPRA program on cervical cancer screening in China either in terms of the screening rate or socioeconomic inequality.

In this study, we analyzed two waves of provincially representative data from China's National Health Services Survey (NHSS) (14) in 2013 and 2018 collected in Jilin Province of China to identify the temporal changes and socioeconomic inequalities in cervical cancer screening in northeastern China.

## Methods

### Study design and data sources

In this study, we analyzed data from two waves of the NHSS of northeastern China collected from Jilin Province in China during the 2 years of 2013 and 2018, respectively. The NHSS of China is a survey administered every 5 years by the Center for Health Statistics and Information of the National Health Commission. The survey is designed to investigate the status of population health, health services demand and utilization, health insurance coverage, medical costs, expenditures, and their financial burden on Chinese residents. NHSS data were collected from a nationally representative sample of Chinese residents following a design of multi-stage stratified random cluster sampling *via* one-to-one interviews using a structured questionnaire. The overall response rate to the NHSS was >90% in both the 2013 and 2018 waves. This study included data from female participants aged 15–64 years old surveyed in the two waves of 2013 and 2018 in Jilin Province of northeastern China and excluded the participants who had any missing values in independent variables. The total sample size of this study, combining the participants surveyed in 2013 and 2018, was 11,616 people. After excluding participants with missing values, a total of 11,611 participants were eligible for inclusion in our data analysis.

### Study procedures and variables

#### Dependent variable

The primary dependent variable in this study was the use of cervical cancer screening during the past 12 months by NHSS participants. In the NHSS, female participants were asked about their use of cervical cancer screening by a question: “Have you received any cervical smear test in the past 12 months?” in 2013 and by a substitute question “Have you received cervical cancer screening (including cervical smear test, liquid-based cytology [LBC] test, or HPV DNA test) in the past 12 months?” in 2018. As mentioned, there are four cervical cancer screening tests: the conventional visual inspection with acetic acid, the pap smear test, the LBC test (15), and the newly introduced HPV deoxyribonucleic acid (DNA) test (2). LBC and HPV testing were introduced for cervical cancer screening in China in 1999 (16). Therefore, the slight difference between the questions between the 2 years was because of the availability of new cervical cancer testing technologies and changes in cervical cancer screening guidelines in China, in that LBC testing and HPV DNA testing were included in 2018 but not 2013 for cervical cancer screening.

#### Independent variables

In this study, we included the following variables as independent variables: residence (rural or urban), age (15–21, 22–29, 30–39, 40–49, 50–59, or 60–64 years old), educational attainment (primary school or below, secondary school, or college and above), travel time to health care facilities (<15, 15–30, or ≥30 mins), parity (0 or ≥1), ethnicity (Han majority or another minority), marital status (married, unmarried, or other), and health insurance (17, 18) [none, Urban Employee Basic Medical Insurance (UEBMI), Urban Resident Basic Medical Insurance (URBMI), New Rural Cooperative Medical Scheme (NRCMS), or other]. According to China's current insurance system (17, 18), the UEBMI scheme covers eligible urban employees and consists of a pooled fund for inpatient care and individual medical savings account for outpatient visits. The URBMI scheme covers the rest of the urban population who are not eligible for enrollment in the UEBMI. The NRCMS is designed to cover all rural populations, and it is financed by the premiums of those enrolled and generous subsidies from both central and local governments. We also included the annual per-capita household income as a proxy for the financial status of participants. We defined five household income categories based on quartiles of annual household income per capita [Q1, <US dollars (USD) $1,005.3; Q2, USD$1,005.3–$1,587.3; Q3, USD$1,587.3–$2,380.9; Q4, USD$2,380.9–$3,703.7; Q5, >USD$3,703.7]. Note that the average of the exchange rate during 2013 and 2018 was as follows: 1 USD = 6.3 yuan (CNY) or the people's renminbi (RMB). The annual per-capita household income in 2013 was adjusted by a cumulative consumer price index rate of 9.3% from 2013 to 2018, which was reported by China's National Bureau of Statistics (http://www.stats.gov.cn/).

### Statistical analysis

A descriptive analysis was conducted to represent the socioeconomic and other characteristics of the study population. The results were also stratified by the year of the NHSS survey and by the participants who underwent cervical cancer screening. Pearson's chi-squared test was performed to compare the distribution of the participants across the categories defined by these characteristics. To compare two proportions between the two survey years, a z-test was performed. A multivariate logistic regression analysis was conducted to determine the factors associated with cervical cancer screening. Separate multivariate logistic regression analyses were performed to examine these associations in each of the two survey years. All statistical analyses were performed in Stata version 11.0 (StataCorp LLC, College Station, TX, USA).

## Results

This study enrolled a total of 11,611 female participants aged 15–64 years old, including 5,490 surveyed in 2013 and 6,121 surveyed in 2018, respectively. Socioeconomic and other characteristics for all participants and those who underwent cervical cancer screening are presented in Table 1. Among all participants, 7,096 participants (61.11%) were from rural areas (3,413 in 2013, 3,683 in 2018), whereas 4,515 participants (38.89%) were from urban areas (2,077 in 2013, 2,438 in 2018). Significant improvements in annual household income per capita (χ^2^ statistic = 292.87, *p* < 0.001) and educational attainment (χ^2^ statistic = 30.06, *p* < 0.001) were observed among all survey participants from 2013 to 2018. The participants surveyed in 2018 tended to be older than those surveyed in 2013 (χ^2^ statistic = 105.31, *p* < 0.001). Health insurance coverage distribution was different between 2013 and 2018 (χ^2^ statistic = 347.64, *p* < 0.001), with the coverage rate increasing from 90.95% to 95.33%. Travel time to health care facilities decreased over time among the participants (χ^2^ statistic = 235.47, *p* < 0.001), and <10% of participants required >15 minutes to reach their closest health care facility. There was a rise in parity over time (χ^2^ statistic = 31.92, *p* < 0.001), but the marital status did not show a significant change from 2013 to 2018.

**Table 1:** Socioeconomic and other characteristics of all study participants and those who underwent cervical cancer screening in 2013 and 2018.

**Characteristic**	**All participants**			**Participants who underwent cervical cancer screening**			**Proportion of participants who underwent cervical cancer screening**	
	**2013, n (%)**	**2018, n (%)**	***p-*value**	**2013, n (%)**	**2018, n (%)**	***p*-value**	**2013, %**	**2018, %**
**Total**	5,490	6,121		652	1,016		11.88%	16.60%
**Residence**			0.027			0.180		
Rural	3,413 (62.17%)	3,683 (60.17%)		290 (44.48%)	486 (47.83%)		8.50%	13.20%
Urban	2,077 (37.83%)	2,438 (39.83%)		362 (55.52%)	530 (52.17%)		17.43%	21.74%
**Annual household income per capita (USD)**			<0.001			<0.001		
Q1: <$1,005.3	1,026 (18.69%)	1,297 (21.19%)		81 (12.42%)	152 (14.96%)		7.89%	11.72%
Q2: $1,005.3–$1,587.3	1,069 (19.47%)	1,260 (20.58%)		98 (15.03%)	142 (13.98%)		9.17%	11.27%
Q3: $1,587.3–$2,380.9	1,396 (25.43%)	1,019 (16.65%)		144 (22.09%)	164 (16.14%)		10.32%	16.09%
Q4: $2380.9–$3703.7	1,198 (21.82%)	1,049 (17.14%)		174 (26.69%)	174 (17.13%)		14.52%	16.59%
Q5: ≥$3,703.7	801 (14.59%)	1,496 (24.44%)		155 (23.77%)	384 (37.80%)		19.35%	25.67%
**Educational attainment**			<0.001			0.005		
Primary school or below	1,493 (27.19%)	1,865 (30.47%)		90 (13.80%)	184 (18.11%)		6.03%	9.87%
Secondary school	3,269 (59.54%)	3,336 (54.50%)		403 (61.81%)	548 (53.94%)		12.33%	16.43%
College or above	728 (13.26%)	920 (15.03%)		159 (24.39%)	284 (27.95%)		21.84%	30.87%
**Age (years)**			<0.001			<0.001		
15–21	287 (5.23%)	260 (4.25%)		0 (0.00%)	3 (0.30%)		0.00%	1.15%
22–29	688 (12.53%)	474 (7.74%)		78 (11.96%)	58 (5.71%)		11.34%	12.24%
30–39	961 (17.50%)	1,127 (18.41%)		162 (24.85%)	281 (27.6%)		16.86%	24.93%
40–49	1,503 (27.38%)	1,593 (26.03%)		237 (36.35%)	337 (33.17%)		15.77%	21.16%
50–59	1,489 (27.12%)	1,882 (30.75%)		142 (21.78%)	274 (26.97%)		9.54%	14.56%
60–64	562 (10.24%)	785 (12.82%)		33 (5.06%)	63 (6.20%)		5.87%	8.03%
**Health insurance**			<0.001			<0.001		
None	497 (9.05%)	286 (4.67%)		42 (6.44%)	24 (2.36%)		8.45%	8.39%
UEBMI	1,064 (19.38%)	941 (15.37%)		223 (34.20%)	258 (25.39%)		20.96%	27.42%
URBMI	875 (15.94%)	897 (14.65%)		136 (20.86%)	123 (12.11%)		15.54%	13.71%
NRCMS	2,794 (50.89%)	3,175 (51.87%)		198 (30.37%)	392 (38.58%)		7.09%	12.35%
Other	260 (4.74%)	822 (13.43%)		53 (8.13%)	219 (21.56%)		20.38%	26.64%
**Travel time to healthcare facilities**			<0.001			<0.001		
<15 mins	4,408 (80.29%)	5,526 (90.26%)		525 (80.52%)	957 (94.19%)		11.91%	17.32%
15–30 mins	616 (11.22%)	365 (5.96%)		87 (13.34%)	46 (4.53%)		14.12%	12.60%
≥30 mins	466 (8.49%)	231 (3.77%)		40 (6.13%)	13 (1.28%)		8.58%	5.63%
**Parity**			<0.001			0.810		
0	771 (14.04%)	649 (10.60%)		43 (6.60%)	64 (6.30%)		5.58%	9.86%
≥1	4,719 (85.96%)	5,472 (89.40%)		609 (93.40%)	952 (93.70%)		12.91%	17.40%
**Ethnicity**			<0.001			0.492		
Han majority	4,961 (90.36%)	5,652 (92.34%)		579 (88.80%)	913 (89.86%)		11.67%	16.15%
Minority	529 (9.64%)	469 (7.66%)		73 (11.20%)	103 (10.14%)		13.80%	21.96%
**Marital status**			0.345			0.239		
Married	4,652 (84.74%)	5,225 (85.36%)		606 (92.94%)	928 (91.34%)		13.03%	17.76%
Unmarried or other	838 (15.26%)	896 (14.64%)		46 (7.06%)	88 (8.66%)		5.49%	9.82%

NRCMS, New Rural Cooperative Medical Scheme; other, government agency health insurance plan or private health insurance plan; UEBMI, Urban Employee Basic Medical Insurance; URBMI, Urban Resident Basic Medical Insurance.

**Figure 1:**
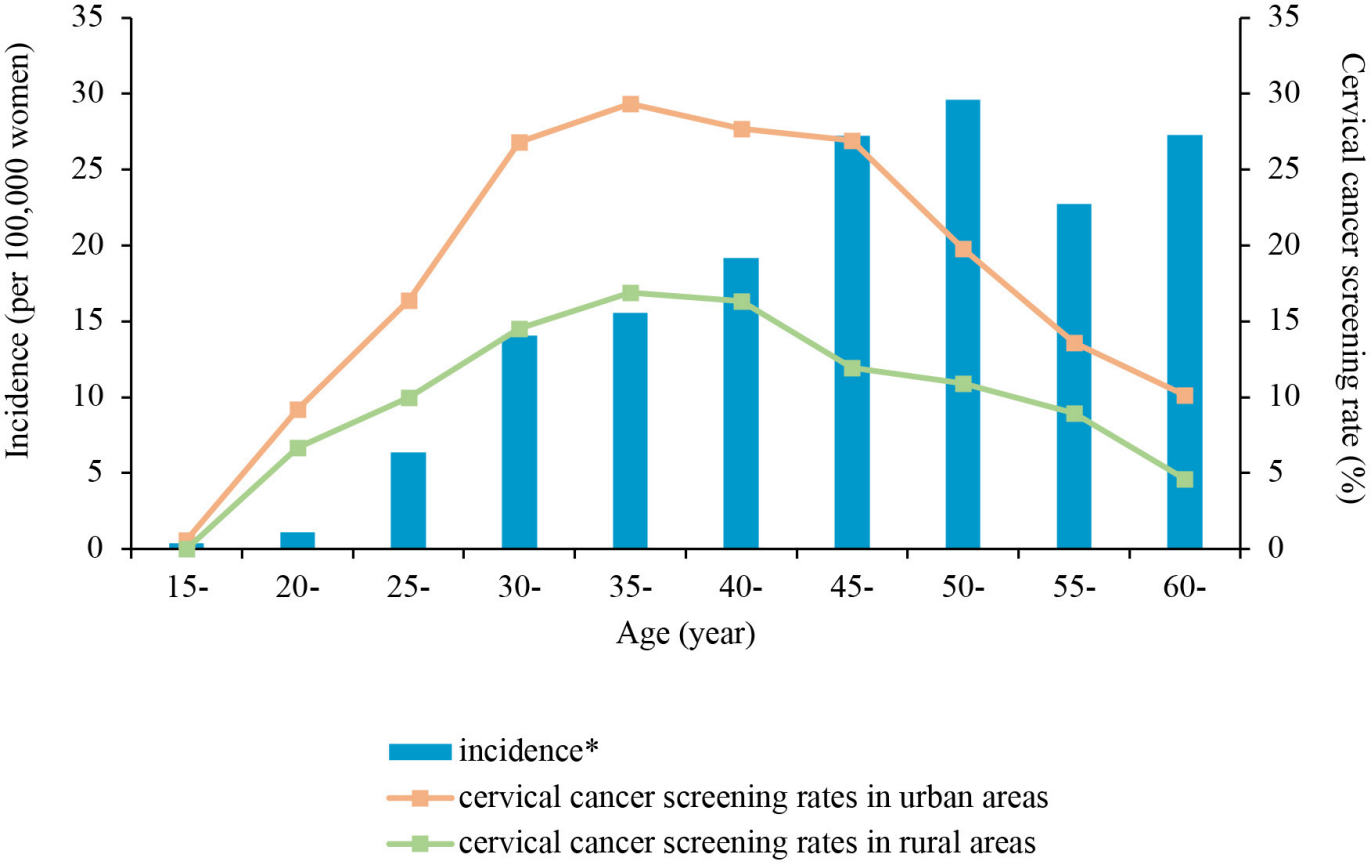
Incidence of cervical cancer reported by the Global Burden of Disease Study 2017 and the 2013 and 2018 total cervical cancer screening rate in Jilin Province of China by age groups and urban-rural areas. *The data of cervical cancer incidence reported by the Global Burden of Disease Study 2017 (33).

Among the participants who underwent cervical cancer screenings, 53.48% in total came from urban areas; more specifically, this percentage was 55.52% in 2013 and 52.17% in 2018, respectively, which did not show a significant difference (χ^2^ statistic = 1.80, *p* = 0.180; Figure 1). The annual household income per capita increased from 2018 to 2013 (χ^2^ statistic = 51.30, *p* < 0.001) among these participants. There was a significant difference in health insurance as well. Travel time to health care facilities decreased over time (χ^2^ statistic = 76.53, *p* < 0.001), while parity, ethnicity, and marital status did not demonstrate any change from 2018 to 2013. The proportion of participants who underwent cervical cancer screening increased significantly from 11.88% in 2013 to 16.60% in 2018 (χ^2^ statistic = 52.47, *p* < 0.001). This shows a significant increase in the use of cervical cancer screening by the population; moreover, the proportion significantly improved in both rural (χ^2^ statistic = 40.16, *p* < 0.001) and urban (χ^2^ statistic = 13.14, *p* < 0.001) areas.

Table 2 reports the odds ratios (ORs) and their corresponding 95% confidence intervals (CIs) along with *p*-values that were obtained from fitting the multivariate logistic regression of the use of cervical cancer screening on the socioeconomic and other factors for the years of 2013 and 2018, respectively, and for all participants taking the year of the survey as an independent variable. The rate of cervical cancer screening increased from 2013 to 2018 (OR: 1.06; 95% CI: 1.04–1.09, *p* < 0.001). Overall, participants from urban areas were more likely to undergo cervical cancer screening tests than those from urban areas (OR: 1.20; 95% CI: 1.03–1.39, *p* = 0.020) when participants in the two survey waves were analyzed as a whole group. However, though this rural-urban inequality was observed in 2013 (OR: 1.34; 95% CI: 1.04–1.73, *p* = 0.022), it disappeared in 2018 (OR: 1.08; 95% CI: 0.89–1.32, *p* = 0.446). The participants in the highest category of annual household income (Q5, >USD$3,703.7) had greater odds of undergoing cervical cancer screening compared to those in the lowest category of annual household income (Q1, <USD$1,005.3) (OR: 1.30; 95% CI: 1.07–1.56, *p* = 0.007); otherwise, there was no significant difference between the income categories.

**Table 2:** Odds ratios, 95% confidence intervals, and *p* values obtained from multivariate logistic regression of the use of cervical cancer screening on the socioeconomic and other factors for 2013 and 2018 in China.

**Characteristic**	**2013**		**2018**		**Total**	
	**OR (95% CI)**	***p*-value**	**OR (95% CI)**	***p*-value**	**OR (95% CI)**	***p*-value**
**Year**						
2013	–	–	–	–	Reference	
2018	–	–	–	–	1.06 (1.04–1.09)	<0.001
**Residence**						
Rural	Reference		Reference		Reference	
Urban	1.34 (1.04–1.73)	0.022	1.08 (0.89–1.32)	0.446	1.20 (1.03–1.39)	0.020
**Annual household income per capita (USD)**						
Q1: <$1,005.3	Reference		Reference		Reference	
Q2: $1,005.3–$1,587.3	0.92 (0.66–1.27)	0.599	0.81 (0.63–1.04)	0.099	0.86 (0.70–1.04)	0.124
Q3: $1,587.3–$2,380.9	0.98 (0.72–1.32)	0.872	1.10 (0.85–1.41)	0.472	1.00 (0.82–1.20)	0.961
Q4: $2,380.9–$3,703.7	1.25 (0.93–1.68)	0.145	0.97 (0.75–1.26)	0.816	1.04 (0.86–1.25)	0.714
Q5: ≥$3,703.7	1.44 (1.05–1.98)	0.026	1.33 (1.04–1.69)	0.021	1.30 (1.07–1.56)	0.007
**Educational attainment**						
Primary school or below	Reference		Reference		Reference	
Secondary school	1.38 (1.06–1.80)	0.019	1.49 (1.22–1.81)	<0.001	1.45 (1.23–1.70)	<0.001
College or above	1.83 (1.27–2.64)	0.001	2.40 (1.82–3.17)	<0.001	2.16 (1.73–2.69)	<0.001
**Age (years)**						
15–21	–	–	0.21 (0.06–0.72)	0.013	0.16 (0.05–0.51)	0.002
22–29	3.76 (2.28–6.18)	<0.001	1.43 (0.94–2.17)	0.098	2.17 (1.59–2.97)	<0.001
30–39	3.90 (2.54–5.98)	<0.001	2.98 (2.19–4.06)	<0.001	3.24 (2.53–4.16)	<0.001
40–49	3.91 (2.61–5.86)	<0.001	2.72 (2.02–3.65)	<0.001	3.09 (2.43–3.92)	<0.001
50–59	1.86 (1.24–2.80)	<0.001	1.97 (1.47–2.64)	<0.001	1.94 (1.53–2.45)	<0.001
60–64	Reference		Reference		Reference	
**Health insurance**						
None	Reference		Reference		Reference	
UEBMI	2.49 (1.71–3.60)	<0.001	2.60 (1.63–4.16)	<0.001	2.42 (1.82–3.23)	<0.001
URBMI	1.95 (1.34–2.84)	0.001	1.49 (0.92–2.40)	0.104	1.63 (1.21–2.19)	0.001
NRCMS	1.03 (0.69–1.54)	0.887	1.78 (1.14–2.78)	0.011	1.37 (1.03–1.82)	0.031
Other	2.78 (1.76–4.39)	<0.001	2.88 (1.82–4.58)	<0.001	2.63 (1.95–3.55)	<0.001
**Travel time to healthcare facilities**						
<15 mins	Reference		Reference		Reference	
15–30 mins	1.51 (1.17–1.99)	0.002	0.83 (0.60–1.14)	0.242	1.17 (0.96–1.42)	0.130
≥30 mins	1.08 (0.76–1.53)	0.656	0.43 (0.24–0.77)	0.005	0.75 (0.56–1.01)	0.056
**Parity**						
0	Reference		Reference		Reference	
≥1	1.89 (1.20–2.98)	0.006	1.41 (1.00–2.01)	0.053	1.62 (1.23–2.14)	0.001
**Ethnicity**						
Han majority	Reference		Reference		Reference	
Minority	1.13 (0.86–1.49)	0.388	1.12 (0.88–1.43)	0.345	1.14 (0.95–1.36)	0.164
**Marital status**						
Married	1.62 (1.10–2.38)	0.015	1.35 (1.02–1.80)	0.034	1.45 (1.15–1.81)	0.001
Unmarried or other	Reference		Reference		Reference	

CI, confidence interval; NRCMS, New Rural Cooperative Medical Scheme; OR, odds ratio; other, government agency health insurance plan or private health insurance plan; UEBMI, Urban Employee Basic Medical Insurance; URBMI, Urban Resident Basic Medical Insurance.

Participants with greater educational attainment were more likely to undergo cervical cancer screening than those with lower educational attainment (*p* < 0.001 for the two categories of “secondary school” and “college and above”), and this trend existed in both 2013 and 2018. Participants who had any type of health insurance were also more likely than those who were not covered by any health insurance to undergo cervical cancer screening tests (*p* < 0.001 for UEBMI, URBMI, and others; *p* = 0.031 for NRCMS). However, exceptions were noted in the group of NRCMS (OR: 1.30; 95% CI: 0.69–1.54, *p* = 0.887) in 2013 and in the group of URBMI in 2018 (OR: 1.49; 95% CI: 0.92–2.40, *p* = 0.104), respectively. The impact of travel time to a health care facility on the rate of cervical cancer screening was insignificant overall (OR: 0.75; 95% CI: 0.56–1.01, *p* = 0.056) and inconsistent from 2013 to 2018.

In total, the rate of cervical cancer screening was higher among the four age groups of participants aged 22–59 years (*p* < 0.001) compared to the reference group of participants aged 60–64 years. However, the rate was lower among participants aged 15–21 years (OR: 0.16; 95% CI: 0.05–0.51, *p* = 0.022; Figure 1). The rate of cervical cancer screening was also significantly associated with parity (OR: 1.62; 95% CI: 1.23–2.41, *p* = 0.001) and marital status (OR: 1.45; 95% CI: 1.15–1.81, *p* = 0.001) but not ethnicity (OR: 1.41; 95% CI: 0.95–1.36, *p* = 0.164).

## Discussion

Using two waves of provincially representative data from China's NHSS in 2013 and 2018 collected in Jilin Province of China, we investigated the temporal changes and socioeconomic inequalities in cervical cancer screening in northeastern China. Our analysis showed that the cervical cancer screening coverage rate improved between 2013 and 2018 in both rural and urban areas, but the overall screening rate in Jilin Province remained far below the target 70% screening rate proposed by the WHO. Although the screening rate in rural areas was still lower than that in urban areas, the rural–urban inequality that was observed in 2013 disappeared in 2018. However, socioeconomic inequality still existed between the highest and the lowest income categories. Women with greater educational attainment or with health insurance were more likely to undergo cervical cancer screening. Our findings suggest that the use of cervical cancer screening still has a big gap to cross before achieving the target and that inequality persists especially socioeconomic inequality.

The WHO set the 70% goal of cervical cancer screening coverage, while the outline of “Healthy China 2030” proposed that the cervical cancer screening rate should reach 80% in 2030 (19). We found that the current screening coverage in China is far below both targets, which may be due to the different definitions of utilization. The WHO goal focuses on the coverage of twice-lifetime screening, while the target in “Healthy China 2030” focuses on screening utilization in the past 5 years. However, our study focused on utilization in the past 12 months, which may underestimate the use of cervical cancer screening.

Comparing the coverage of cervical cancer screening in Jilin Province to that of other provinces in China, we found that the overall screening rate was similar among women older than 18 years old reported in 2010 (12, 13). However, a study by You et al. (20) in Jiangsu Province using data from the NHSS reported that, in 2013, coverage of cervical cancer screening was 35.57%. Coverage in Jiangsu Province was higher in the study of You et al. maybe because only women aged 36–65 years old were included, while our study enrolled women older than 15 years old. In some developed countries, such as Norway (6), the coverage rate may reach >70% after the nationwide screening program is carried out. In South Africa and Turkey, the rates of cervical cancer screening were found to be 52.0% (21) and 22.0% (22), respectively. The coverage of cervical cancer screening in our study is lower than the target and lower than the level in some developed countries.

The use of cervical cancer screening increased from 2013 to 2018 in both rural and urban areas. Although the cervical cancer screening rate was lower in rural areas, the rural-urban inequality that existed in 2013 disappeared in 2018. This result may be explained using different screening strategies in urban and rural areas. Organized screening programs provide free screening services to all eligible women in rural areas so that, no matter a woman's household income or type of social health insurance, she can receive free service equally (11). However, in urban areas, women who cannot obtain organized screening need to search for services at a hospital. Under this circumstance, vulnerable women in urban areas are more likely to be influenced by socioeconomic factors, and, finally, the rural-urban inequality vanished.

We found that people with the highest income status had the greatest rate of cervical cancer screening in 2013 and 2018. This finding might be partly due to the low capacity to pay despite the service provided by the NCCSPRA being free. Socioeconomic inequalities attributed to a low capacity to pay were widely reported as barriers to universal coverage (20, 23). Income-related inequality is common all over the world and is documented in 67 countries (24). A study also found that a 20% increase in outpatient reimbursement could increase the rate of cervical cancer screening by 2.3% (25). A previous study in Korea found that, after Korea's National Cancer Screening Program expanded free cancer screening to people in the lower 50% of household income bracket in 2005, the disparity in Korea was improved and only the highest income group showed a significant difference compared to the lowest income group (26). Our result might reflect the importance of an organized program in eliminating income inequality.

Apart from the low capacity to pay, a reduced willingness to undergo screening and less health awareness was also associated with the utilization of screening services (27) in our study. The coverage showed an increasing trend as the level of educational attainment increased from 2013 to 2018. Women with greater educational attainment were more likely to undergo cervical cancer screening, which may be attributed to women with lower educational attainment not realizing the importance of cervical cancer screening (28). This finding was in concordance with those of studies from both developing and developed countries documenting that the screening rate of cervical cancer among women with greater educational attainment was higher (22, 26, 27, 29, 30). Studies reported that organized programs implemented in Denmark and Sweden did not eliminate the inequalities associated with educational attainment (31, 32), matching with our result. We found that organized screening programs in rural areas could not eliminate the inequalities caused by educational attainment, even though coverage among women with lower educational attainment grew faster.

Besides socioeconomic inequality, age also is an important indicator that caused inequality in screening rates. The latest data from the Global Burden of Disease study (33) showed that, with advancing age, the risk of cervical cancer increases (Figure 1). The incidence of cervical cancer was highest among women aged >45 years old, while the cervical cancer screening rate was highest among women aged 30–49 years and dropped significantly after 50 years of age. The demand for cervical cancer screening differed greatly from its actual utilization (33). This phenomenon may be due to the misunderstanding of menopause, in that older women believe menopause can reduce the risk of cervical cancer (34). Instead, the capability of menopause to reduce the risk of cervical cancer is a misconception, which had a negative impact on screening participation (35). Based on the available evidence, the incidence of cancer among women aged 50–64 years with adequate screening was 1/6 that among those not screened (36). In implementing an organized screening program, special attention should be paid and targeted policies should be designed for elderly women in response to the growing disease burden.

The cervical cancer incidence in China in 2017 was 15.8 per 100,000 women, and the WHO's goal of reducing the cervical cancer incidence was set to <4 per 100,000 women. These study findings indicate that, to reach the 70% target put forward by the WHO and eliminate the inequalities, the effectiveness of organized screening programs in rural areas should be continually improved. Moreover, organized screening programs should also be implemented in urban areas and carried out using a multi-sector strategy to cover the whole process, including mobilization and monitoring.

Well-run organized screening programs should integrate health education, service provision, staff training, and effective monitoring. Before providing services, physicians and communities should mobilize women to improve their health awareness and therefore enhance their willingness to undergo screening. The efficacy of organized screening in rural areas still requires monitoring and enhancement to ensure an increase in coverage and the effectiveness of screening programs. It is important to include the whole process, from mobilization to effect monitoring, into an organized screening program. Based on the current disease burden, organized screening programs should involve policies targeting older women and should pay special attention to vulnerable populations (e.g., those with less educational achievements and a lower socioeconomic status).

Our study has inherited limitations from the design of the NHSS survey. First, the language of the survey question on whether or not the participant underwent any cervical cancer screening tests in 2013 and 2018 was not consistent. While it is possible to have underestimated the use of cervical cancer screening services in 2013, we thought that the difference in questions was mainly due to the use of new technologies and changes in cervical cancer screening guidelines, as mentioned above. Thus, it would have little effect on our conclusion. In addition, the cervical cancer screening utilization indicator in the NHSS survey was self-reported, and this may introduce a certain degree of recall bias. Another limitation of our research is that the socioeconomic characteristics in our analysis only included a limited number of subject-level factors: residence, annual household income, education, health insurance status, access to healthcare facilities, ethnicity, and marital status. Therefore, our investigation does not cover all aspects of socioeconomic domains and therefore is not comprehensive. Further studies are needed to conduct a comprehensive study on cervical cancer screening.

## Conclusions

Cervical cancer screening coverage was improved from 2013 to 2018 in northeastern China but remained far below the target screening rate of 70% proposed by the WHO. Although the rural-urban inequality disappeared, other socioeconomic inequalities remained. Our findings suggest that an organized program may help to increase equality. However, the use of cervical cancer screening alone may not resolve the issues in achieving a high targeted rate and reducing the socioeconomic inequality of cervical cancer screening.

## Data availability statement

The data that support the findings of this study are available from the National Health Services Survey of the Chinese Center for Disease Control and Prevention, but restrictions apply to the availability of these data, which were used under license for the current study, and so are not publicly available. Data are however available from the authors upon reasonable request from the corresponding author and with permission of the Chinese Center for Disease Control and Prevention.

## Author contributions

The authors confirm contribution to the article as follows: study conception and design and draft manuscript preparation: YL, JG, GZ, BZ, and XF. Analysis and interpretation of results: YL, JG, BZ, and XF. All authors reviewed the results and approved the final version of the manuscript.

## Conflict of interest

The authors declare that the research was conducted in the absence of any commercial or financial relationships that could be construed as a potential conflict of interest.

## Publisher's note

All claims expressed in this article are solely those of the authors and do not necessarily represent those of their affiliated organizations, or those of the publisher, the editors and the reviewers. Any product that may be evaluated in this article, or claim that may be made by its manufacturer, is not guaranteed or endorsed by the publisher.
